# Access to alcohol reduction websites facilitated by primary care practices: a potential solution to the "know/do" gap in primary care

**DOI:** 10.1186/1940-0640-7-S1-A88

**Published:** 2012-10-09

**Authors:** Paul Wallace, Leo Pas, Pierluigi Struzzo

**Affiliations:** 1 Department of Primary Care and Population Health, University College London, London, UK; 2 Primary Health Care European Project on Alcohol, Domus Medica, Wezembeek Oppem, Belgium; 3 Regional Center for Training in Primary Care, Monfalcone, Italy

## 

In primary health-care (PHC) settings, less than 10% of hazardous and harmful drinkers are identified, and less than 5% of those who could benefit are offered brief interventions (BIs). Delivery of BI adds up to 15 minutes to the primary consultation, constituting a significant barrier to implementation by PHC professionals. A review of trials of computer-based interventions (on- and off-line) for college drinkers found them to be more effective than no treatment and as effective as alternative treatment approaches. The recent large-scale trial of the online Down Your Drink (DYD) intervention found potentially significant reductions in alcohol consumption and risky drinking behavior, but there was evidence that users of the DYD website made only relatively limited use of it (average, 2.33 visits). Facilitated access by professionals in PHC settings could not only increase patient engagement but also address the "know/do” gap in PHC by removing the need for providers to deliver a time-consuming face-to-face intervention. The University College of London eHealth team has undertaken an exploratory qualitative study using interviews and participant observation of facilitated access to DYD at PHC practices in two London localities (Kingston and Islington) to assess acceptability and uptake. Preliminary results suggest good levels of acceptability and uptake of facilitated access to DYD. Findings are presented together with proposals for a noninferiority international randomized controlled trial to determine whether there is similarity in outcomes in patients identified as risky drinkers who receive facilitated access to an alcohol reduction website and those who receive conventional face-to-face BIs. Research is needed to determine whether PHC-based facilitated access to alcohol reduction websites can reduce the "know/do" gap in primary-care alcohol screening and intervention.

